# Analysis of More than 16,000 Human Tumor and Normal Tissues Identifies Uroplakin 3B as a Useful Diagnostic Marker for Mesothelioma and Normal Mesothelial Cells

**DOI:** 10.3390/diagnostics12102516

**Published:** 2022-10-17

**Authors:** Maximilian Lennartz, Dennis Atug, Sebastian Dwertmann Rico, Viktor Reiswich, Florian Viehweger, Franziska Büscheck, Martina Kluth, Claudia Hube-Magg, Andrea Hinsch, Christian Bernreuther, Guido Sauter, Eike Burandt, Andreas H. Marx, Till Krech, Ronald Simon, Sarah Minner, Till S. Clauditz, Frank Jacobsen, Patrick Lebok, Natalia Gorbokon, Katharina Möller, Stefan Steurer, Christoph Fraune

**Affiliations:** 1 Department of Pathology, University Medical Center Hamburg-Eppendorf, 20246 Hamburg, Germany; 2 Department of Pathology, Academic Hospital Fuerth, 90766 Fuerth, Germany; 3 Department of Pathology, Clinical Center Osnabrueck, 49076 Osnabrueck, Germany

**Keywords:** uroplakin 3B, immunohistochemistry, tissue micro array, mesothelioma, diagnostic

## Abstract

Uroplakin 3B (Upk3b) is involved in stabilizing and strengthening the urothelial cell layer of the bladder. Based on RNA expression studies, Upk3b is expressed in a limited number of normal and tumor tissues. The potential use of Upk3b as a diagnostic or prognostic marker in tumor diagnosis has not yet been extensively investigated. A tissue microarray containing 17,693 samples from 151 different tumor types/subtypes and 608 samples of 76 different normal tissue types was analyzed by immunohistochemistry. In normal tissues, Upk3b expression was largely limited to mesothelial cells, urothelial umbrella cells, and amnion cells. In tumor tissues, Upk3b was detectable in only 17 of 151 (11.3%) of tumor types. Upk3b expression was most frequent in mesotheliomas (82.1% of epithelioid and 30.8% of biphasic) and in urothelial tumors of the urinary bladder, where the positivity rate decreased from 61.9% in pTaG2 (low grade) to 58.0% in pTaG3 (high grade) and 14.6% in pT2-4 cancers. Among pT2-4 urothelial carcinomas, Upk3b staining was unrelated to tumor stage, lymph node status, and patient prognosis. Less commonly, Upk3b expression was also seen in Brenner tumors of the ovary (10.8%), as well as in four other subtypes of ovarian cancer (0.9–10.6%). Four additional tumor entities showed a weak to moderate Upk3b positivity in less than 5% of cases. In summary, Upk3b immunohistochemistry is a useful diagnostic tool for the distinction of mesotheliomas from other thoracic tumors and the visualization of normal mesothelial and umbrella cells.

## 1. Introduction

Uroplakin 3B (Upk3b) is one out of five known uroplakin proteins (Upk) that jointly form “apical asymmetrical unit membrane (AUM) plaques”. These structures stabilize epithelial cells that are exposed to physical extension. AUM plaques enable the urothelium to considerably stretch during bladder distension and prevent urothelial cells from rupturing [1,2,3]. All Upks are assembled in the endoplasmic reticulum (ER). Upk3b heterodimerizes with Upk1b before it is released from the ER [3,4]. Upk heterodimers later constitute tetramers and concentric hexameric rings which are eventually integrated into the cell surface membrane [3,5]. Several Upk proteins have also been suggested to exert further functional effects with a role in signal transduction, cell development, growth, and motility [6,7,8,9].

Although the biology and function of Upk3b is well understood, there is much less certainty with respect to the normal and neoplastic tissue distribution of the protein. Rudat et al. described Upk3b expression in urothelium and in mesothelial cells under normal conditions and demonstrated that Upk3b-deficient mice did not show morphologic or molecular abnormalities in differentiation or integrity of these tissues [7]. RNA screening databases including a wide range of normal tissues [10,11,12,13] have also described Upk3b expression in lungs, esophagus, salivary glands, testes, and organs of the female genital tract, but most of these organs are lined by normal mesothelium, which may have affected these analyses. Data on a potential role of Upk3b in cancer are so far solely derived from RNA screening studies. The TCGA database describes Upk3b RNA expression to occur in urothelial, ovarian, endometrial, cervical, stomach, and lung cancers (Cancer Genome Atlas Research Network, https://www.cancer.gov/about-nci/organization/ccg/research/structural-genomics/tcga, accessed on 10 August 2022). Studies analyzing Upk3b protein expression in cancer by immunohistochemistry (IHC) are so far lacking.

To comprehensively determine the protein expression pattern and the potential diagnostic utility of Upk3b IHC, a large-scale study on normal human tissues and human neoplasms is needed. In this study, we therefore analyzed a preexisting set of tissue microarrays (TMAs) containing more than 17,000 tumor tissue samples from 151 different tumor types and subtypes, as well as 76 non-neoplastic tissue categories for Upk3b expression by IHC.

## 2. Materials and Methods

**Tissue Microarrays (TMAs).** Our normal tissue TMA was composed of 8 samples from 8 different donors for each of 76 different normal tissue types (608 samples on one slide). The cancer TMAs included a total of 17,693 primary tumors from 151 different tumor types and subtypes. Detailed histopathological data were available for 1,663 and clinical outcome information for 254 urinary bladder cancer patients. The median follow-up time was 14 months (range: 1–77 months) for overall survival, 11 months (range: 1–75 months) for recurrence-free survival, and 14.5 months (range: 1–77 months) for cancer-specific survival. The composition of normal and cancer TMAs is described in the results section. All samples were from the archives of the Department of Pathology, University Hospital of Hamburg, Germany, the Department of Pathology, Clinical Center Osnabrueck, Germany, and the Department of Pathology, Academic Hospital Fuerth, Germany. Tissues were fixed in 4% buffered formalin and then embedded in paraffin. The TMA manufacturing process has been previously described in detail [14,15]. In brief, one tissue spot (diameter: 0.6 mm) was transmitted from a cancer containing donor block to an empty recipient paraffin block. The use of archived remnants of diagnostic tissues for TMA manufacturing, their analysis for research purposes, and the use of patient data were according to local laws (HmbKHG, §12), and analysis had been approved by the local ethics committee (Ethics commission Hamburg, WF-049/09). All work has been carried out in compliance with the Helsinki Declaration.

**Immunohistochemistry (IHC).** Freshly prepared TMA sections were immunostained on one day and in one experiment. Slides were deparaffinized with xylol, rehydrated through a graded alcohol series, and exposed to heat-induced antigen retrieval for 5 min in an autoclave at 121 °C in pH 7.8 Dako Target Retrieval Solution™ (Agilent, CA, USA). Endogenous peroxidase activity was blocked with Dako Peroxidase Blocking Solution™ (Agilent, CA, USA; #52023) for 10 min. Primary antibody specific for Upk3b (mouse monoclonal, MSVA-736M, #4879-736M, MS Validated Antibodies GmbH, Hamburg, Germany) was applied at 37 °C for 60 min at a dilution of 1:150. For the purpose of antibody validation, the normal tissue TMA was also analyzed by the mouse monoclonal Upk3b antibody C362 (#ab237778, Abcam, Cambridge, United Kingdom) at a dilution of 1:150 and an otherwise identical protocol. Bound antibody was visualized using the EnVision Kit™ (Agilent, CA, USA; #K5007) according to the manufacturer’s directions. The sections were counterstained with haemalaun. All stained slides were manually analyzed by one pathologist (SS). In normal tissues, Upk3b stained cell types were identified, and their staining intensity was estimated (1+ = weak staining, 2+ = moderate staining, 3+ = strong staining). Normal cell types without detectable staining were designated as “negative”. In tumor tissues, the percentage of tumor cells with detectable staining and the prevailing staining level in these tumor cells (1+, 2+, 3+) were estimated. For statistical analyses, the staining results were categorized into four groups. Tumors without any staining were considered negative. Tumors with 1+ staining intensity in ≤70% of tumor cells or 2+ intensity in ≤30% of tumor cells were considered weakly positive. Tumors with 1+ staining intensity in >70% of tumor cells, 2+ intensity in 31–70% of tumor cells, or 3+ intensity in ≤30% of tumor cells were regarded moderately positive. Tumors with 2+ intensity in >70% of tumor cells or 3+ intensity in >30% of tumor cells were considered strongly positive.

**Statistics.** Statistical calculations were performed with JMP 16 software (SAS Institute Inc., Wake County, NC, USA). Contingency tables and the chi²-test were performed to search for associations between Upk3b and tumor phenotype. Survival curves were calculated according to Kaplan-Meier. The Log-Rank test was applied to detect significant differences between groups. A *p*-value of ≤0.05 was considered to be statistically significant.

## 3. Results

### 3.1. Technical Issues

A total of 16,185 (91.5%) of 17,693 tumor samples were interpretable in our tumor TMA analysis. Non-interpretable samples demonstrated a lack of unequivocal tumor cells or loss of the tissue spot during technical procedures. A sufficient number of samples (≥4) of each normal tissue type was evaluable.

### 3.2. Upk3b in Normal Tissue

**Figure 1:**
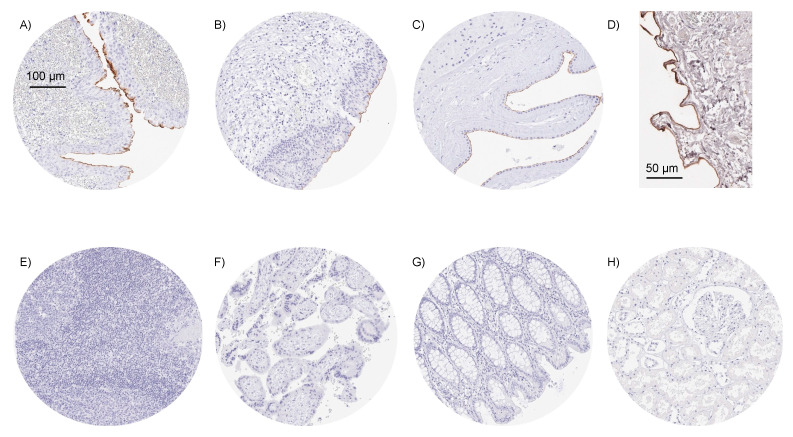
**Examples of Upk3b immunostaining in selected normal tissues**. A strong apical membranous Upk3b positivity is seen in umbrella cells of urothelium of the renal pelvis (**A**), umbrella cells of the urinary bladder urothelium (**B**), amnion cells of the placenta (**C**), and of mesothelial cells of the peritoneum (**D**). Upk3b staining is absent in tissues from tonsil (**E**), first trimenon placenta (**F**), colon mucosa (**G**), and the renal parenchyma (**H**).

**Figure 2:**
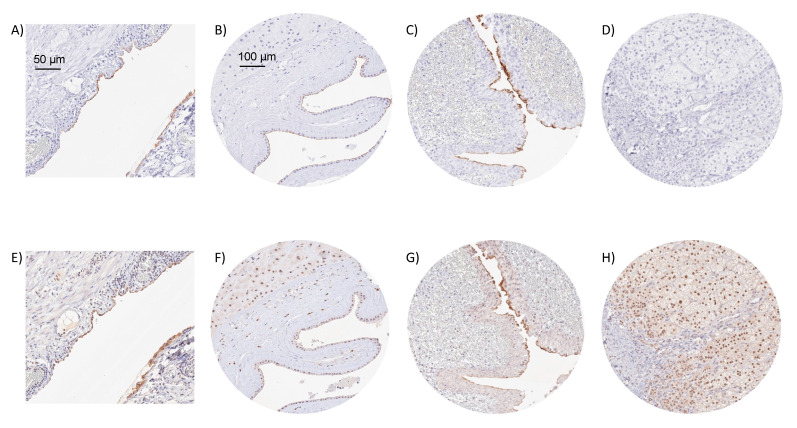
**IHC validation by comparison of antibodies.** Using MSVA-736M, an apical membranous Upk3b positivity is seen in mesothelial cells covering an appendix (**A**), amnion cells of a placenta (**B**), and umbrella cells of the renal pelvis urothelium (**C**), while staining is absent in adrenal gland (**D**). Using clone C362, a similar membranous staining is seen in mesothelial cells of the appendix (**E**), amnion cells (**F**), and urothelial umbrella cells (**G**), despite of a higher level of background staining. Clone C362 also results in a significant nuclear staining of adrenocortical cells (**H**) which is not seen by MSVA-736M.

Using the monoclonal mouse antibody MSVA-736M, a strong Upk3b staining was seen in umbrella cells of the urothelium, normal mesothelial cells, and amnion cells. In all three cell types, staining was particularly strong at the apical/luminal cell membrane and often even limited to this region. Representative images of normal tissues are shown in Figure 1. Upk3b staining was not observed (by MSVA-736M) in squamous epithelium, sebaceous glands, gastrointestinal epithelium, Brunner glands, gall bladder, liver, pancreas, salivary glands, breast, endocervix, endometrium, fallopian tube, ovary, respiratory epithelium, lung, kidney, testis, thyroid, parathyroid, adrenal gland, hypophysis, or the brain. Only at higher concentrations (1:50) MSVA-736M resulted in a probably non-specific staining of the muscular wall or perivascular fibrous structures of small vessels. The mouse monoclonal anti-Upk3b antibody C362 was used for validation of MSVA-736M findings (Figure 2). C362 resulted in considerably higher background staining and nuclear positivity in several tissue types but confirmed the membranous Upk3b staining of urothelial, mesothelial, and amnion cells.

### 3.3. Upk3b in Cancer

Upk3b immunostaining in tumors was predominantly membranous. It was detectable in 356 (2.2%) of the 16,185 analyzable tumors, including 283 (1.7%) with weak, 39 (0.2%) with moderate, and 34 (0.2%) with strong immunostaining. The highest rates of Upk3b positivity were seen in epithelioid (82.1%) and biphasic mesotheliomas (30.8%; positivity always limited to epithelioid cells), followed by various categories of urothelial tumors (10.8–45.7%) including Brenner tumors of the ovary (10.8%) as well as four other subtypes of ovarian cancers (0.9–10.6%). Four additional tumor entities showed a weak to moderate Upk3b positivity in less than 5% of cases (Table 1).

**Figure 3:**
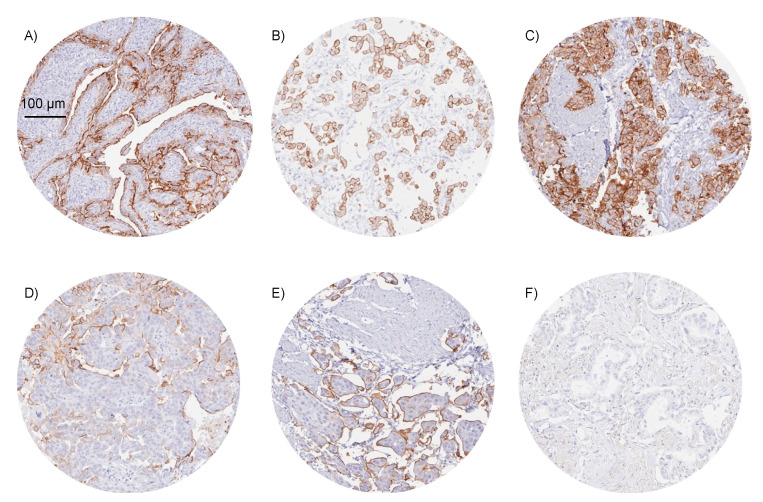
**Examples of Upk3b immunostaining in selected cancers**. The panels show a strong membranous Upk3b staining in samples of two epithelioid mesotheliomas (**A**,**B**), and a muscle-invasive urothelial carcinoma of the urinary bladder (**C**). Upk3b staining is more focal and predominantly seen on surface membranes in a serous high-grade ovarian carcinoma (**D**) and an invasive micropapillary urothelial cancer (**E**). Upk3b staining is absent in an adenocarcinoma of the lung (**F**).

Representative images of Upk3b positive tumors are shown in Figure 3.

**Figure 4:**
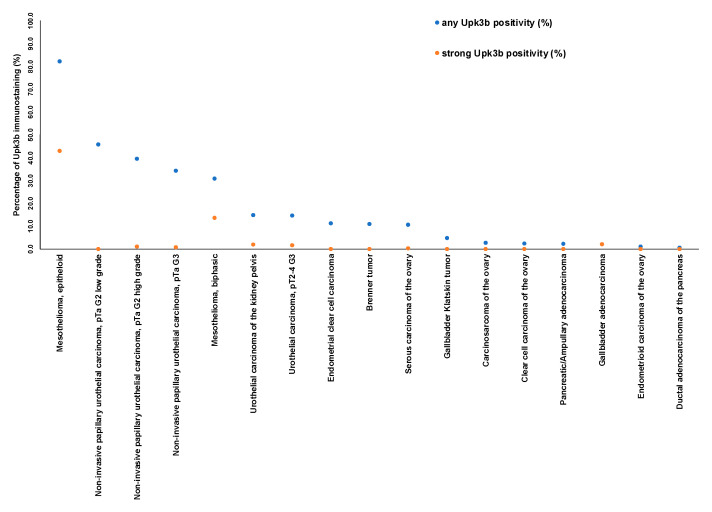
**Ranking of cancers according to the fraction of Upk3b positive cases.** Both the percentage of positive cases (blue dots) and the percentage of strongly positive cases (orange dots) are shown.

A graphical representation of a ranking order of Upk3b positive and strongly positive cancers is given in Figure 4.

Within non-invasive urothelial neoplasms of the urinary bladder, the rate of Upk3b positive cases decreased from 61.9% in pTaG2 (low grade) to 58.0% in pTaG3 (high grade, *p* < 0.05, Table 2).

**Figure 5:**
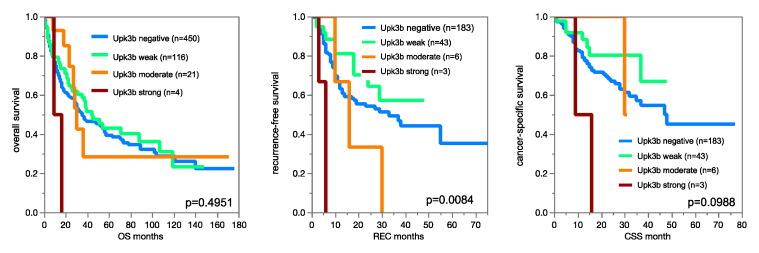
Impact of Upk3b immunostaining on overall survival, recurrence-free survival, and cancer specific survival in muscle-invasive urothelial carcinomas.

The fraction of Upk3b positive cases further decreased to 14.6% in muscle invasive urothelial carcinomas (*p* < 0.0001). Within muscle-invasive urothelial cancers, Upk3b immunostaining was unrelated to pT, pN, and patient prognosis (Figure 5).

## 4. Discussion

Our normal tissue analysis revealed that Upk3b expression was largely limited to normal mesothelial cells, umbrella cells, and amnion cells. Given the function and location of these cell types, our observation fits well with the presumed function of Upk3b as a stabilizer of cell layers that periodically undergo distension. Our findings are in contrast, however, to data from RNA expression databases suggesting a larger range of Upk3b positive normal tissues. In these databases, 23 of 51 analyzed normal tissue categories showed Upk3b expression [10,11,12,13]. It seems possible that mainly technical issues account for this discrepancy. Normal tissue is a complex mixture of different cell types, and RNA extraction requires disintegration of the tissue sample. We assume that Upk3b RNA might firstly originate from the mesothelial cells that cover most of the tissues rated Upk3b positive in these databases. This ubiquitous Upk3b RNA expression might have contributed to a lack of interest in this protein. As for June 2022, we are not aware of any study using IHC for Upk3b protein analysis in human cancer samples. Our tumor analysis identified Upk3b expression in only 17 of 151 analyzed cancer categories and enabled the definition of a ranking order of tumor types according to their UpK3b positivity rate which essentially paralleled the findings in normal tissues. The most frequently positive cancers included epithelioid (82.1%) and biphasic (30.8%) mesotheliomas, six subtypes of urothelial neoplasms including Brenner tumors of the ovary (10.8–45.7%), as well as a four other subtypes of ovarian cancers (0.9–10.6%). Four further tumor entities contained <5% of cases with a (mostly weak) Upk3 positivity. These findings are essentially in agreement with RNA expression data available from The Cancer Genome Atlas PanCancer project (see Figure S1) which had identified highest rates and levels of Upk3b expression in urothelial and ovarian tumors.

The comparative analysis of different tumor entities identified two potential diagnostic applications of Upk3b IHC: (a) distinction of malignant mesotheliomas from adenocarcinomas of the lung and metastatic adenocarcinomas to the lung, and (b) distinction of urothelial neoplasms from other tumors invading the urinary bladder. Both diagnostic challenges are common in surgical pathology and usually require panels of antibodies for a reliable distinction [16,17]. In our data, the particularly high positivity rate of Upk3b in epithelioid mesotheliomas (82.1%) in combination with the complete absence of Upk3b in pulmonary adenocarcinomas (0/184) and in other adenocarcinomas that are known to frequently metastasize to the lung, such as prostate cancer (0/491), breast cancer (0/706), as well as colorectal (0/2483), gastric (0/474), and pancreatic adenocarcinomas (0/734) permits the use of Upk3b as a putative diagnostic marker for the difficult distinction of malignant mesothelioma from primary or metastatic adenocarcinomas in the lung. Biomarkers that are most commonly used for this purpose include mesothelial (calretinin, D2-40, WT1 and cytokeratin 5/6), epithelial (Claudin 4), and adenocarcinoma markers (Ber-EP4, TTF-1, CEA, MOC31, Napsin A) [18]. Studies are now needed to determine the relative diagnostic utility of Upk3b IHC in comparison to already established markers.

It is of note that a 2+ or 3+ positivity of >80% of tumor cells was largely limited to mesotheliomas (11.1–42.9%). Apart from mesotheliomas, such a staining pattern was only seen in 1.3% of 1037 analyzed urothelial cancers, 0.2% of 539 serous high-grade carcinomas of the ovary, and 2% of 50 adenocarcinomas of the gallbladder. Given these findings and the constantly high Upk3b expression in normal mesothelial cells, one would expect that virtually every cancer metastasizing to the pleural or abdominal cavity should result in a significant fraction of Upk3b negative cancerous cells if Upk3b IHC was applied to effusion specimens. Therefore, Upk3b IHC, which should label all normal mesothelial cells, might facilitate cancer cell detection in diagnostic cytology.

The relatively high rate of Upk3b positivity in ovarian carcinomas may be reflective of the close morphological and functional similarity of ovarian surface epithelium (also referred to as “ovarian mesothelium”) and mesothelial cells which regularly express Upk3b [19]. It has been proposed that distinct subsets of serous ovarian cancers may be derived from either ovarian surface epithelium or epithelial cells of the fimbria of the fallopian tube [20,21]. Based on our data, it might be speculated that Upk3b expression might potentially distinguish these subtypes and predominantly occur in cancers derived from the ovarian surface epithelium. It is of note that Upk3b positivity was often strong in ovarian cancer cells but usually involved rather small subsets of cells which resulted in a staining classification of “weak” or “moderate” positivity according to our predefined criteria. A role of mesothelial cells that might be intermingled between ovarian cancer cells has recently been proposed as a mechanism for epithelial-mesenchymal transition and metastasis of ovarian carcinomas [22,23]. However, scattered Upk3b positive cells morphologically always resembled typical cancer cells and not mesothelial cells in our tumors. Irrespective of the origin and role of Upk3b positive ovarian cancer cells, their occurrence is a limitation for using Upk3b IHC for diagnosing malignant mesothelioma of the peritoneum. A metastatic ovarian carcinoma to the lung should also be considered in case of a Upk3b positive thoracic tumor although this clinical scenario is highly uncommon as the first manifestation of an ovarian carcinoma [24].

Although urothelial neoplasms comprise 68.7% of all Upk3b positive cases in our study, the utility of Upk3b for the distinction of urothelial cancer from other neoplasms may be limited. High positivity rates of >60% were only seen in non-invasive papillary carcinomas while almost 80% of the muscle-invasive cancers which are causing most diagnostic difficulties were Upk3b negative. Due to of the limitation of Upk3b immunostaining to umbrella cells in normal urothelium, Upk3b positivity of urothelial neoplasms is mostly driven by Upk3b expressing remnants of the umbrella cell layer which are predominantly observed in low grade non-invasive papillary carcinomas. As such, Upk3b IHC emerges as a suitable tool to selectively visualize the umbrella cell layer, the integrity of which has been suggested as a criterion for the grading and classification of papillary urothelial neoplasms [25]. In our daily routine, we utilize Upk3b IHC for the distinction of umbrella cells from dysplastic urothelial cells in biopsies containing a flat urothelium including only few cell layers. For this purpose, Upk3b is better suited than cytokeratin 20 (CK20) because—in contrast to CK20—Upk3b is less prone to stain dysplastic urothelium.

Given the large-scale of our study, emphasis was placed on a thorough validation of our assay. The International Working Group for Antibody Validation (IWGAV) has proposed that antibody validation for IHC on formalin fixed tissues should include either a comparison of the findings obtained by two different independent antibodies or a comparison with expression data obtained by another independent method [26]. The limited concordance of our IHC results with RNA data obtained in three independent RNA screening studies, including the Human Protein Atlas (HPA) RNA-seq tissue dataset [13], the FANTOM5 project [10,11], and the Genotype-Tissue Expression (GTEx) project [12] can be explained by mesothelial cells that are possibly included in tissues samples of lung, esophagus, adipose tissue, fallopian tube, ovary, vagina, prostate, or testis. All these tissues have been reported as Upk3b RNA positive in the literature but were always Upk3b negative by our IHC analysis. In order to ensure that any potential antibody cross reactivity would be detected in our validation experiment as much as possible, 76 different normal tissues categories were analyzed by two independent antibodies. The fact that all stains identified by MSVA-736M were confirmed by the antibody C362 underlines the validity of our results. At the same time, the “comparison of antibodies” approach revealed various cross reactivities of C362, mostly involving cell nuclei and a tendency of MSVA-736M to cross-react with perivascular fibers in case of overconcentration.

In summary, our data provide a comprehensive overview of Upk3b protein expression in normal and neoplastic human tissues. The expression pattern of Upk3b in normal and neoplastic tissues offers potential diagnostic applications of Upk3b IHC, including the distinction of mesotheliomas from other primary or metastatic thoracic tumors as well as the visualization of normal mesothelial and umbrella cells.

## Figures and Tables

**Table 1:** Upk3 immunostaining in human tumors. int. = interpretable, neg. = negative, mod. = moderate, str. = strong.

			Upk3b Immunostaining Result				
	Tumor Entity	on TMA (n)	int. (n)	neg. (%)	weak (%)	mod. (%)	str. (%)
**Tumors of the skin**	Pilomatrixoma	35	33	100.0	0.0	0.0	0.0
	Basal cell carcinoma	89	79	100.0	0.0	0.0	0.0
	Benign nevus	29	28	100.0	0.0	0.0	0.0
	Squamous cell carcinoma of the skin	145	142	100.0	0.0	0.0	0.0
	Malignant melanoma	65	62	100.0	0.0	0.0	0.0
	Malignant melanoma lymph node metastasis	86	78	100.0	0.0	0.0	0.0
	Merkel cell carcinoma	48	43	100.0	0.0	0.0	0.0
**Tumors of the head and neck**	Squamous cell carcinoma of the larynx	109	107	100.0	0.0	0.0	0.0
	Squamous cell carcinoma of the pharynx	60	59	100.0	0.0	0.0	0.0
	Oral squamous cell carcinoma (floor of the mouth)	130	129	100.0	0.0	0.0	0.0
	Pleomorphic adenoma of the parotid gland	50	49	100.0	0.0	0.0	0.0
	Warthin tumor of the parotid gland	104	104	100.0	0.0	0.0	0.0
	Adenocarcinoma, NOS (Papillary Cystadenocarcinoma)	14	13	100.0	0.0	0.0	0.0
	Salivary duct carcinoma	15	15	100.0	0.0	0.0	0.0
	Acinic cell carcinoma of the salivary gland	181	160	100.0	0.0	0.0	0.0
	Adenocarcinoma NOS of the salivary gland	109	92	100.0	0.0	0.0	0.0
	Adenoid cystic carcinoma of the salivary gland	180	116	100.0	0.0	0.0	0.0
	Basal cell adenocarcinoma of the salivary gland	25	23	100.0	0.0	0.0	0.0
	Basal cell adenoma of the salivary gland	101	96	100.0	0.0	0.0	0.0
	Epithelial-myoepithelial carcinoma of the salivary gland	53	53	100.0	0.0	0.0	0.0
	Mucoepidermoid carcinoma of the salivary gland	343	307	100.0	0.0	0.0	0.0
	Myoepithelial carcinoma of the salivary gland	21	21	100.0	0.0	0.0	0.0
	Myoepithelioma of the salivary gland	11	10	100.0	0.0	0.0	0.0
	Oncocytic carcinoma of the salivary gland	12	12	100.0	0.0	0.0	0.0
	Polymorphous adenocarcinoma, low grade, of the salivary gland	41	37	100.0	0.0	0.0	0.0
	Pleomorphic adenoma of the salivary gland	53	43	100.0	0.0	0.0	0.0
**Tumors of the lung, pleura, and thymus**	Adenocarcinoma of the lung	196	184	100.0	0.0	0.0	0.0
	Squamous cell carcinoma of the lung	80	75	100.0	0.0	0.0	0.0
	Small cell carcinoma of the lung	16	16	100.0	0.0	0.0	0.0
	Mesothelioma, epithelioid	40	28	17.9	25.0	14.3	42.9
	Mesothelioma, biphasic	77	52	69.2	11.5	5.8	13.5
	Thymoma	29	29	100.0	0.0	0.0	0.0
**Tumors of the female genital tract**	Squamous cell carcinoma of the vagina	78	73	100.0	0.0	0.0	0.0
	Squamous cell carcinoma of the vulva	157	147	100.0	0.0	0.0	0.0
	Squamous cell carcinoma of the cervix	136	129	100.0	0.0	0.0	0.0
	Adenocarcinoma of the cervix	23	23	100.0	0.0	0.0	0.0
	Endometrioid endometrial carcinoma	338	296	100.0	0.0	0.0	0.0
	Endometrial serous carcinoma	86	75	100.0	0.0	0.0	0.0
	Carcinosarcoma of the uterus	57	44	100.0	0.0	0.0	0.0
	Endometrial carcinoma, high grade, G3	13	11	100.0	0.0	0.0	0.0
	Endometrial clear cell carcinoma	9	9	88.9	11.1	0.0	0.0
	Endometrioid carcinoma of the ovary	130	108	99.1	0.9	0.0	0.0
	Serous carcinoma of the ovary	580	539	89.4	8.7	1.7	0.2
	Mucinous carcinoma of the ovary	101	81	100.0	0.0	0.0	0.0
	Clear cell carcinoma of the ovary	51	42	97.6	2.4	0.0	0.0
	Carcinosarcoma of the ovary	47	39	97.4	2.6	0.0	0.0
	Granulosa cell tumor of the ovary	44	43	100.0	0.0	0.0	0.0
	Leydig cell tumor of the ovary	4	4	100.0	0.0	0.0	0.0
	Sertoli cell tumor of the ovary	1	1	100.0	0.0	0.0	0.0
	Sertoli Leydig cell tumor of the ovary	3	3	100.0	0.0	0.0	0.0
	Steroid cell tumor of the ovary	3	3	100.0	0.0	0.0	0.0
	Brenner tumor	41	37	89.2	10.8	0.0	0.0
**Tumors of the breast**	Invasive breast carcinoma of no special type	499	472	100.0	0.0	0.0	0.0
	Lobular carcinoma of the breast	192	168	100.0	0.0	0.0	0.0
	Medullary carcinoma of the breast	23	22	100.0	0.0	0.0	0.0
	Tubular carcinoma of the breast	20	17	100.0	0.0	0.0	0.0
	Mucinous carcinoma of the breast	29	27	100.0	0.0	0.0	0.0
	Phyllodes tumor of the breast	50	41	100.0	0.0	0.0	0.0
**Tumors of the digestive** **system**	Adenomatous polyp, low-grade dysplasia	50	50	100.0	0.0	0.0	0.0
	Adenomatous polyp, high-grade dysplasia	50	48	100.0	0.0	0.0	0.0
	Adenocarcinoma of the colon	2483	2392	100.0	0.0	0.0	0.0
	Gastric adenocarcinoma, diffuse type	215	210	100.0	0.0	0.0	0.0
	Gastric adenocarcinoma, intestinal type	215	204	100.0	0.0	0.0	0.0
	Gastric adenocarcinoma, mixed type	62	60	100.0	0.0	0.0	0.0
	Adenocarcinoma of the esophagus	83	81	100.0	0.0	0.0	0.0
	Squamous cell carcinoma of the esophagus	76	66	100.0	0.0	0.0	0.0
	Squamous cell carcinoma of the anal canal	91	87	100.0	0.0	0.0	0.0
	Cholangiocarcinoma	58	56	100.0	0.0	0.0	0.0
	Gallbladder adenocarcinoma	51	50	98.0	0.0	0.0	2.0
	Gallbladder Klatskin tumor	42	42	95.2	2.4	2.4	0.0
	Hepatocellular carcinoma	312	294	100.0	0.0	0.0	0.0
	Ductal adenocarcinoma of the pancreas	659	641	99.5	0.5	0.0	0.0
	Pancreatic/Ampullary adenocarcinoma	98	93	97.8	2.2	0.0	0.0
	Acinar cell carcinoma of the pancreas	18	17	100.0	0.0	0.0	0.0
	Gastrointestinal stromal tumor (GIST)	62	60	100.0	0.0	0.0	0.0
**Tumors of the urinary system**	Non-invasive papillary urothelial carcinoma, pTa G2 low grade	177	116	54.3	44.0	1.7	0.0
	Non-invasive papillary urothelial carcinoma, pTa G2 high grade	141	104	60.6	34.6	3.8	1.0
	Non-invasive papillary urothelial carcinoma, pTa G3	219	155	65.8	32.9	0.6	0.6
	Urothelial carcinoma, pT2-4 G3	735	608	85.4	10.7	2.3	1.6
	Squamous cell carcinoma of the bladder	22	22	100.0	0.0	0.0	0.0
	Small cell neuroendocrine carcinoma of the bladder	23	23	100.0	0.0	0.0	0.0
	Sarcomatoid urothelial carcinoma	25	24	100.0	0.0	0.0	0.0
	Urothelial carcinoma of the kidney pelvis	62	54	85.2	11.1	1.9	1.9
	Clear cell renal cell carcinoma	1287	1148	100.0	0.0	0.0	0.0
	Papillary renal cell carcinoma	368	323	100.0	0.0	0.0	0.0
	Clear cell (tubulo) papillary renal cell carcinoma	26	24	100.0	0.0	0.0	0.0
	Chromophobe renal cell carcinoma	170	143	100.0	0.0	0.0	0.0
	Oncocytoma	257	217	100.0	0.0	0.0	0.0
**Tumors of the male genital organs**	Adenocarcinoma of the prostate, Gleason 3+3	83	83	100.0	0.0	0.0	0.0
	Adenocarcinoma of the prostate, Gleason 4+4	80	75	100.0	0.0	0.0	0.0
	Adenocarcinoma of the prostate, Gleason 5+5	85	84	100.0	0.0	0.0	0.0
	Adenocarcinoma of the prostate (recurrence)	258	249	100.0	0.0	0.0	0.0
	Small cell neuroendocrine carcinoma of the prostate	19	18	100.0	0.0	0.0	0.0
	Seminoma	682	653	100.0	0.0	0.0	0.0
	Embryonal carcinoma of the testis	54	43	100.0	0.0	0.0	0.0
	Leydig cell tumor of the testis	31	29	100.0	0.0	0.0	0.0
	Sertoli cell tumor of the testis	2	2	100.0	0.0	0.0	0.0
	Sex cord stromal tumor of the testis	1	1	100.0	0.0	0.0	0.0
	Spermatocytic tumor of the testis	1	1	100.0	0.0	0.0	0.0
	Yolk sac tumor	53	43	100.0	0.0	0.0	0.0
	Teratoma	53	18	100.0	0.0	0.0	0.0
	Squamous cell carcinoma of the penis	92	86	100.0	0.0	0.0	0.0
**Tumors of endocrine organs**	Adenoma of the thyroid gland	113	111	100.0	0.0	0.0	0.0
	Papillary thyroid carcinoma	391	372	100.0	0.0	0.0	0.0
	Follicular thyroid carcinoma	154	153	100.0	0.0	0.0	0.0
	Medullary thyroid carcinoma	111	109	100.0	0.0	0.0	0.0
	Parathyroid gland adenoma	43	35	100.0	0.0	0.0	0.0
	Anaplastic thyroid carcinoma	45	42	100.0	0.0	0.0	0.0
	Adrenal cortical adenoma	50	38	100.0	0.0	0.0	0.0
	Adrenal cortical carcinoma	28	28	100.0	0.0	0.0	0.0
	Phaeochromocytoma	50	50	100.0	0.0	0.0	0.0
	Appendix, neuroendocrine tumor (NET)	25	19	100.0	0.0	0.0	0.0
	Colorectal, neuroendocrine tumor (NET)	12	10	100.0	0.0	0.0	0.0
	Ileum, neuroendocrine tumor (NET)	53	51	100.0	0.0	0.0	0.0
	Lung, neuroendocrine tumor (NET)	29	29	100.0	0.0	0.0	0.0
	Pancreas, neuroendocrine tumor (NET)	101	93	100.0	0.0	0.0	0.0
	Colorectal, neuroendocrine carcinoma (NEC)	14	13	100.0	0.0	0.0	0.0
	Ileum, neuroendocrine carcinoma (NEC)	8	8	100.0	0.0	0.0	0.0
	Gallbladder, neuroendocrine carcinoma (NEC)	4	4	100.0	0.0	0.0	0.0
	Pancreas, neuroendocrine carcinoma (NEC)	14	13	100.0	0.0	0.0	0.0
**Tumors of haemotopoetic and lymphoid tissues**	Hodgkin Lymphoma	103	101	100.0	0.0	0.0	0.0
	Small lymphocytic lymphoma, B-cell type (B-SLL/B-CLL)	50	49	100.0	0.0	0.0	0.0
	Diffuse large B cell lymphoma (DLBCL)	113	109	100.0	0.0	0.0	0.0
	Follicular lymphoma	88	84	100.0	0.0	0.0	0.0
	T-cell non-Hodgkin lymphoma	25	25	100.0	0.0	0.0	0.0
	Mantle cell lymphoma	18	18	100.0	0.0	0.0	0.0
	Marginal zone lymphoma	16	15	100.0	0.0	0.0	0.0
	Diffuse large B-cell lymphoma (DLBCL) in the testis	16	16	100.0	0.0	0.0	0.0
	Burkitt lymphoma	5	4	100.0	0.0	0.0	0.0
**Tumors of soft tissue and bone**	Tendosynovial giant cell tumor	45	45	100.0	0.0	0.0	0.0
	Granular cell tumor	53	44	100.0	0.0	0.0	0.0
	Leiomyoma	50	50	100.0	0.0	0.0	0.0
	Leiomyosarcoma	94	93	100.0	0.0	0.0	0.0
	Liposarcoma	145	139	100.0	0.0	0.0	0.0
	Malignant peripheral nerve sheath tumor (MPNST)	15	15	100.0	0.0	0.0	0.0
	Myofibrosarcoma	26	26	100.0	0.0	0.0	0.0
	Angiosarcoma	74	67	100.0	0.0	0.0	0.0
	Angiomyolipoma	91	91	100.0	0.0	0.0	0.0
	Dermatofibrosarcoma protuberans	21	19	100.0	0.0	0.0	0.0
	Ganglioneuroma	14	13	100.0	0.0	0.0	0.0
	Kaposi sarcoma	8	6	100.0	0.0	0.0	0.0
	Neurofibroma	117	99	100.0	0.0	0.0	0.0
	Sarcoma, not otherwise specified (NOS)	74	72	100.0	0.0	0.0	0.0
	Paraganglioma	41	40	100.0	0.0	0.0	0.0
	Ewing sarcoma	23	20	100.0	0.0	0.0	0.0
	Rhabdomyosarcoma	7	7	100.0	0.0	0.0	0.0
	Schwannoma	122	112	100.0	0.0	0.0	0.0
	Synovial sarcoma	12	11	100.0	0.0	0.0	0.0
	Osteosarcoma	44	39	100.0	0.0	0.0	0.0
	Chondrosarcoma	40	27	100.0	0.0	0.0	0.0
	Rhabdoid tumor	5	5	100.0	0.0	0.0	0.0

**Table 2:** Upk3b immunostaining and tumor phenotype.

	Upk3b Immunostaining Result					
	n	Negative (%)	Weak (%)	Moderate (%)	Strong (%)	*p*
all cancers	2638	64.1	30.9	3.9	1.1	
pTa G2 low	430	38.1	54.7	6	1.2	0.0055
pTa G2 high	209	38.8	54.5	5.7	1	
pTa G3	138	42	58	0	0	
pT2	419	74.9	19.1	4.3	1.7	0.0244
pT3	551	76.2	19.6	3.4	0.7	
pT4	274	77	20.4	2.2	0.4	
G2 *	116	70.7	22.4	6	0.9	0.4355
G3 *	1524	75.5	19.8	3.3	1.3	
pN0 *	642	78.3	18.4	2.8	0.5	0.0234
pN+ *	411	70.6	23.4	5.4	0.7	

* Only in the subset of pT2–pT4 cancers, abbreviations: pT: pathological tumor stage, G: grade, pN: pathological lymph node status.

## Data Availability

All data generated or analyzed during this study are included in this published article.

## Supplementary Materials

The following supporting information can be downloaded at: https://www.mdpi.com/article/10.3390/diagnostics12102516/s1, Figure S1: Upk3b mRNA expression in different human cancer types. The plot was generated in September 2022 from the cBioPartal website (http://www.cbioportal.org, accessed on 9 September 2022), selecting “TCGA PanCancer Atlas studies” (32 studies with 10,976 samples), query for “Upk3b“, and plotting “mRNA vs dx“. Linear scale was selected for the vertical axis, and information on mutation type and structural variants as well as copy number was deselected.
