# Supplementary figures and images for: Analysis of More than 16,000 Human Tumor and Normal Tissues Identifies Uroplakin 3B as a Useful Diagnostic Marker for Mesothelioma and Normal Mesothelial Cells
Source: Diagnostics (Basel). 2022 Oct 17;12(10):2516. doi: 10.3390/diagnostics12102516 (PMC9600073; doi:10.3390/diagnostics12102516)

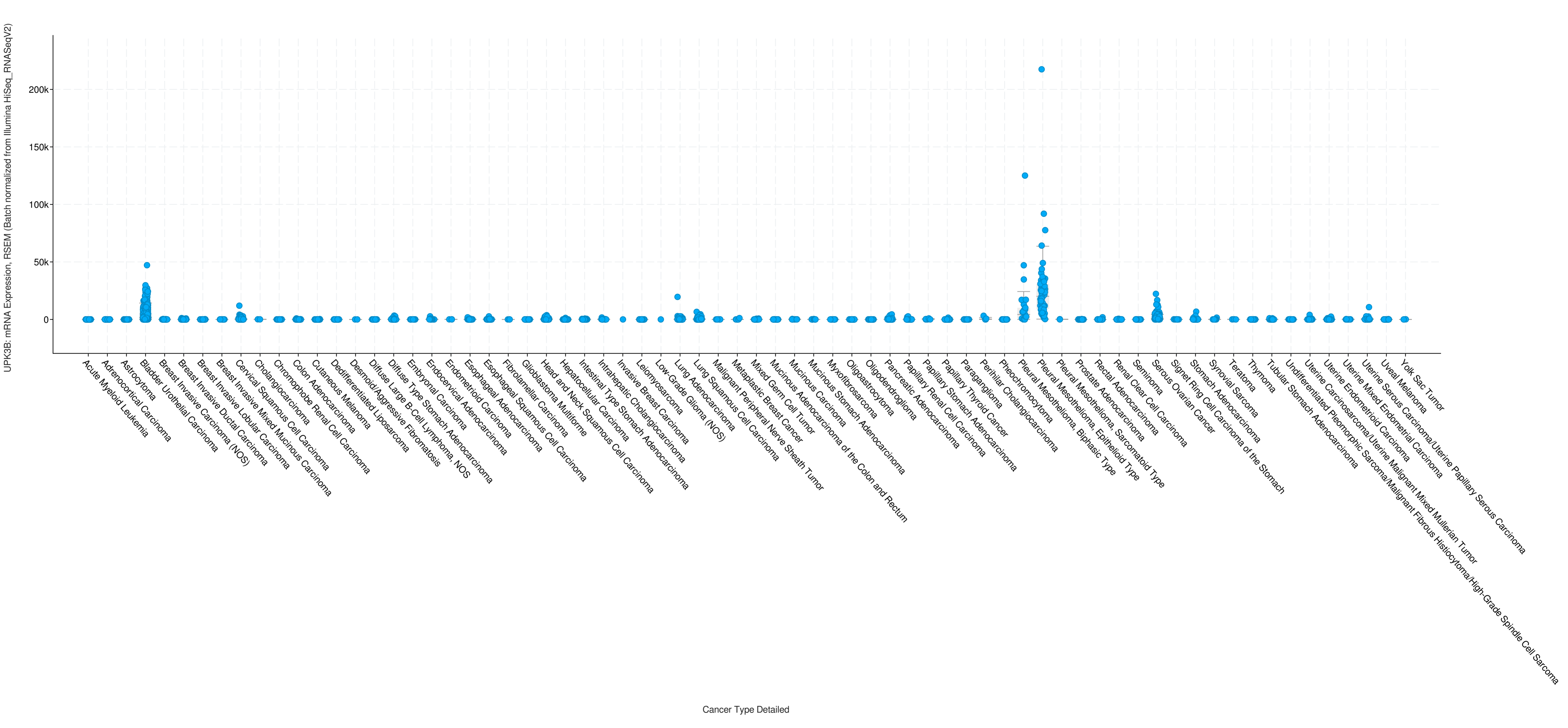

Supplement: Supplementary file 1 [file diagnostics-12-02516-s001.zip › diagnostics-1893467-supplementary.pdf]
